# A programmable epidermal microfluidic valving system for wearable biofluid management and contextual biomarker analysis

**DOI:** 10.1038/s41467-020-18238-6

**Published:** 2020-09-02

**Authors:** Haisong Lin, Jiawei Tan, Jialun Zhu, Shuyu Lin, Yichao Zhao, Wenzhuo Yu, Hannaneh Hojaiji, Bo Wang, Siyang Yang, Xuanbing Cheng, Zhaoqing Wang, Eric Tang, Christopher Yeung, Sam Emaminejad

**Affiliations:** 1 Interconnected & Integrated Bioelectronics Lab (I²BL), Department of Electrical and Computer Engineering, University of California, Los Angeles, Los Angeles, CA USA; 2 Department of Materials Science and Engineering, University of California, Los Angeles, Los Angeles, CA USA; 3 Department of Bioengineering, University of California, Los Angeles, Los Angeles, CA USA

**Keywords:** Biomedical engineering, Mechanical engineering, Materials for devices, Fluidics

## Abstract

Active biofluid management is central to the realization of wearable bioanalytical platforms that are poised to autonomously provide frequent, real-time, and accurate measures of biomarkers in epidermally-retrievable biofluids (e.g., sweat). Accordingly, here, a programmable epidermal microfluidic valving system is devised, which is capable of biofluid sampling, routing, and compartmentalization for biomarker analysis. At its core, the system is a network of individually-addressable microheater-controlled thermo-responsive hydrogel valves, augmented with a pressure regulation mechanism to accommodate pressure built-up, when interfacing sweat glands. The active biofluid control achieved by this system is harnessed to create unprecedented wearable bioanalytical capabilities at both the sensor level (decoupling the confounding influence of flow rate variability on sensor response) and the system level (facilitating context-based sensor selection/protection). Through integration with a wireless flexible printed circuit board and seamless bilateral communication with consumer electronics (e.g., smartwatch), contextually-relevant (scheduled/on-demand) on-body biomarker data acquisition/display was achieved.

## Abstract (web summary)

Wearable biosensors have been used successfully for biomarker analysis, however, a lack of control over sampling limits applications. Here, the authors report a programmable microfluidic valve to control flow rate, sampling times and allow for biofluid routing and compartmentalisation.

## Introduction

Wearable biomarker sensing technologies enable personalized and precision medicine by allowing the frequent, longitudinal, and comprehensive assessment of an individual’s health^1–8^. Recent advances in biochemical sensor development, device fabrication and integration technology, and low-power electronics have paved the path for the realization of wearable epidermal microfluidic systems^4,6^, capable of analyzing epidermally retrievable biofluids^9,10^ (e.g., sweat and interstitial fluid), to access molecular-level biomarker information^11–13^. Previously reported wearable microfluidic biomarker sensors successfully demonstrated electrochemical and colorimetric sensing interfaces for the on-body detection of analytes^1–3^. These sensors rely on the analysis of biofluid samples that are passively collected in predefined microfluidic structures to minimize evaporation^4–6,14,15^. Their lack of active control on biofluid flow fundamentally renders them (1) susceptible to operationally relevant confounders such as flow rate variability, (2) incapable of performing diverse bioanalytical operations (e.g., incubation), and (3) incapable of delivering programmable biofluid management functionalities (e.g., biofluid routing and compartmentalization) that are critical to the operational autonomy of the envisioned systems, such as capturing biomarker readings at contextually relevant timepoints.

To this end, valving is fundamental to active biofluid management, because it enables flow control. The significance of valving has already been demonstrated in microfluidic-based lab-on-a-chip platforms^16–19^. Specifically, programmable valving systems delivered active manipulation and control of small-scale (~nano/microliter) fluid flow within networks of microfluidic channels, forming separated compartments to perform biochemical reactions in an addressable manner^20–22^. Such valving systems were positioned to execute synchronous/asynchronous sequential and parallel fluid manipulation tasks autonomously, leading to the creation of new microfluidic solutions for various applications including diagnostics and -omics^23,24^. To date, such programmable valving systems have not been adapted for integration into lab-on-the-body-like wearable platforms, which is primarily due to the bulkiness of the actuation instruments^25^ (e.g., external mechanical pumps and optical excitation systems). Recently, in the context of wearable platforms, valving interfaces—embedded within sophisticated flexible epidermal microfluidic configurations—were reported, which successfully demonstrated on-body biofluid routing, but they were either passive or required manual mechanical activation^26–28^.

To render active biofluid management in a wearable format, here, we devise an electronically programmable microfluidic valving system, which is capable of biofluid sampling, routing, and compartmentalization for biomarker analysis. The core of the microfluidic system is a network of individually addressable microheater-controlled thermo-responsive poly(N-isopropylacrylamide) (PNIPAM) hydrogel valves. To realize this system, we devise a simple, high-throughput, and low-cost fabrication scheme to develop hydrogel arrays on a tape-based flexible substrate. The fabricated hydrogel arrays can be incorporated within a 3D flexible microfluidic module, following an extensible vertical integration scheme, which allows for the assembly of microfluidic embodiments and actuation/sensing electrode arrays within a compact footprint. To adapt the valving system for on-body biofluid harvesting, specifically, in the context of interfacing with pressure-driven bio-interfaces (e.g., sweat glands), a pressure regulation mechanism is devised, informed by an electronic-hydraulic analogy.

The active fluid control achieved by this system is harnessed to create new wearable bioanalytical capabilities at both the sensor and system levels. At the sensor level, the valving capability is exploited to decouple the confounding influence of flow rate variability on the sensor response, an issue which is well-reported in the context of conventional lab-on-a-chip platforms^29–32^, but overlooked by previously reported wearable sensors. At the system level, valving is leveraged to render addressable biofluid routing and compartmentalization. These capabilities can be positioned to render context-based sensor selection/protection, where the mode of analysis will be selected depending on the user’s need, behavior, and activity.

To deliver seamless control command and biomarker data communication, the sensor array-coupled valving system is interfaced with a custom-developed wireless flexible printed circuit board (FPCB), equipped with multichannel valve actuation and signal-processing capabilities. Through bilateral Bluetooth communication with a smartwatch, preloaded with a custom-designed user interface, biomarker data acquisition, and display at scheduled/on-demand timepoints are achieved. The complete wearable valve-enabled bioanalytical platform was used to take selective biomarker readings, on-body, at various contextually relevant timepoints.

## Results

### Operational principles of the wearable valving system

**Fig. 1:**
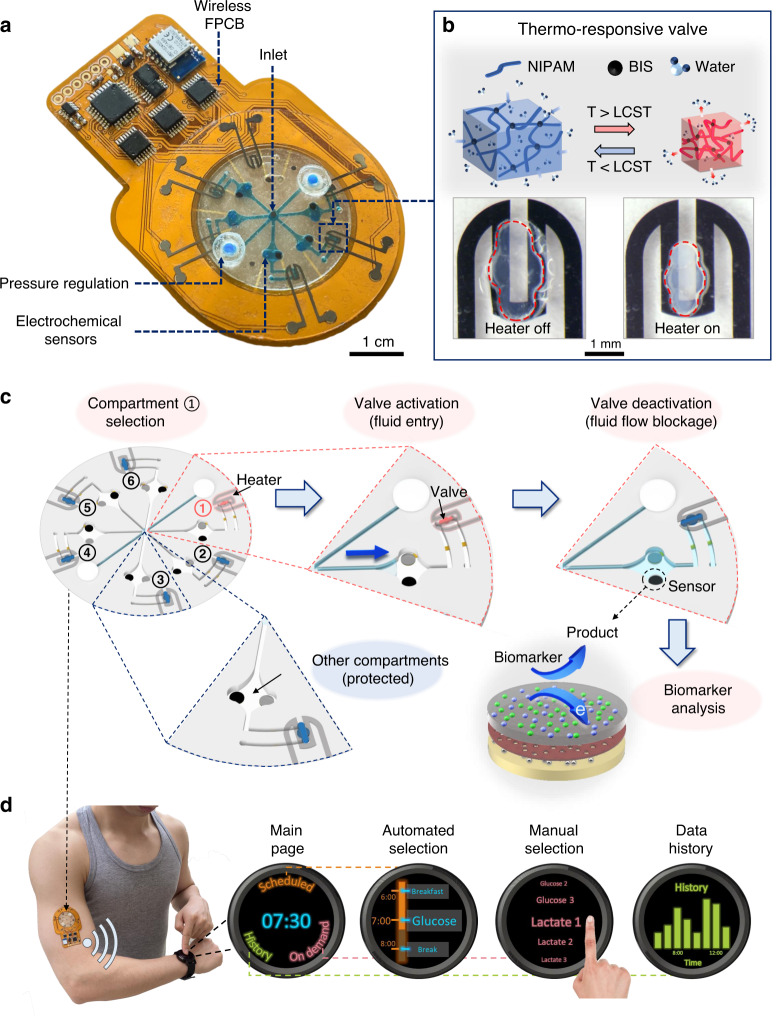
A fully integrated wearable valving system (concept and operational principle). **a** Illustration of a representative wearable bioanalytical platform, consisting of an integrated programmable microfluidic valving system interfacing a FPCB. **b** Illustration of PNIPAM hydrogel shrinkage/expansion in response to temperature change above/below its LCST (induced by activation/deactivation of the microheater). **c** A schematic operation example of the programmable microfluidic valving system, demonstrating biofluid routing, compartmentalization, and analysis in the selected compartment and sensor protection in the nonselected compartments. **d** Illustration of control commands (automated and manual) communication for scheduled and on-demand biomarker data acquisition with the aid of user interfaces preloaded on smart consumer electronics.

Figure 1a illustrates a representative pressure-regulated six-compartment valving system—with a sweat collection inlet at the center and an electrochemical sensing interface within each compartment—interfacing a wireless flexible circuit board to form a fully integrated wearable bioanalytical platform. To construct the valve, we specifically use a PNIPAM-based hydrogel (Fig. 1b and Supplementary Fig. 1a, synthesized from a N-isopropylacrylamide (NIPAM) monomer and *N*,*N*′-methylenebis(acrylamide), BIS crosslinker), which significantly shrinks/expands in response to local temperature increments/decrements, above/below its lower critical solution temperature (LCST)^33^. By embedding this hydrogel within a microfluidic channel, the volumetric thermal responsiveness of the hydrogel can be exploited to effectively permit/block fluid flow via activation/deactivation of the heater. Previous efforts have already demonstrated the utility of thermo-responsive hydrogel-based valving for controlling fluid flow within conventional lab-on-a-chip devices^16,17,21,25^. However, they required manually operated and bulky external instrumentation to actuate the hydrogels, preventing their translation into wearable platforms. Here, we devised a circuit-controlled micropatterned heater (on a flexible substrate) to actuate the hydrogels. In this way, we formed a miniaturized programmable valve, which can be extended into an addressable array, and subsequently, exploited to realize a valve-gated multicompartment bioanalytical platform amenable for wearable applications.

An example operation of our valving system is shown in Fig. 1c. In this example, the valve (downstream of the microfluidic channel) in compartment 1 is first activated (while others remain deactivated) to route and sample biofluid. Then, it is deactivated to block the flow, allowing for biofluid compartmentalization and analysis (using an electrochemical sensor positioned upstream of the channel). Accordingly, sample analysis can be performed—without the confounding influence of flow rate variability—by the sensor(s) in the addressed compartment, while the sensors in the other compartments remain protected.

This addressable compartmentalization capability can be exploited to take biomarker readings at scheduled/on-demand timepoints, thus enabling contextual biomarker analysis. In the presented wearable bioanalytical platform, valve activation and sensor output signal processing are delivered with the aid of a circuit board, which is equipped with a multichannel programmable current source and analog front-end circuits. Through bilateral Bluetooth communication with personal smart electronics (e.g., smartwatch), preloaded with a custom-designed user interface, biomarker data acquisition timepoints (pre-scheduled/on-demand) can be programmed (via automated/manual commands) and biomarker data can be displayed in real-time (Fig. 1d).

### Wearable valve-gated microfluidic networks

For fluid valving, ideally, a binary off/on valve operation is desired, where fluid flow is completely blocked with no leakage in the off-state (when the valve is deactivated), and fluid flow is permitted in the on-state (when the valve is activated). In the context of our thermo-responsive PNIPAM-based hydrogel, off/on transition is achieved upon decreasing/increasing the temperature below/above the LCST. The thermo-responsive property of PNIPAM stems from the coexistence of hydrophilic amide and hydrophobic propyl groups within its polymer structure^34^. When the hydrogel’s temperature is lower than its LCST, the hydrogen-bonding interactions between the amide group and the water molecules are dominant. Therefore, the hydrogel becomes highly hydrated, leading to its structural expansion. Conversely, when the hydrogel’s temperature is higher than its LCST, the hydrogen-bonding interactions become weaker and the interactions between the hydrophobic propyl group and the water molecules are dominant. As a result, the water is released from the hydrogel structure, leading to hydrogel shrinkage.

**Fig. 2:**
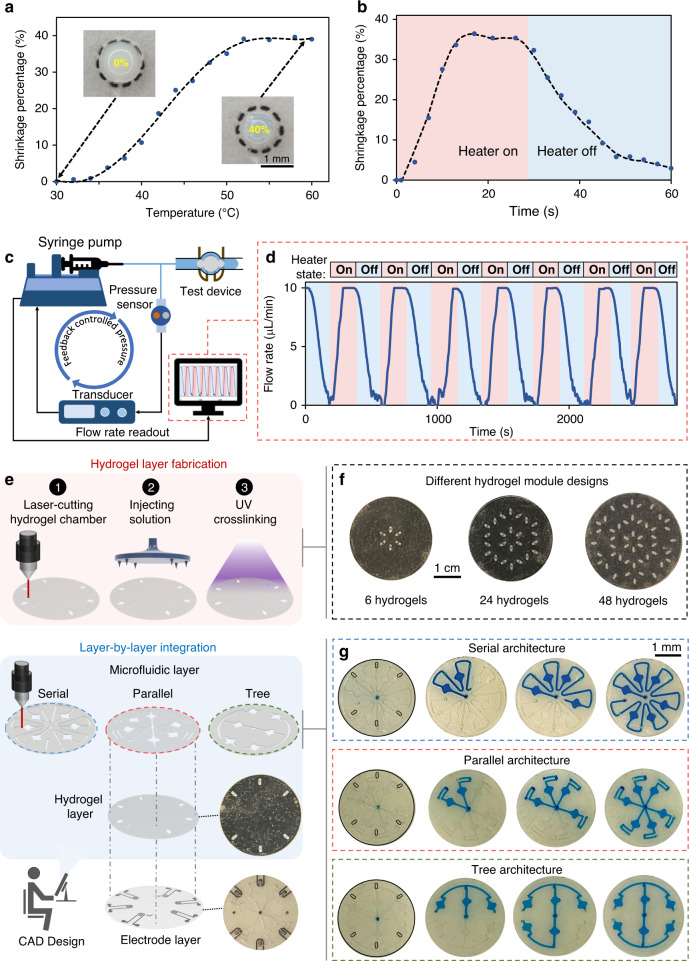
Fabrication and characterization of valve-gated microfluidic networks. **a** Standalone PNIPAM hydrogel shrinkage percentage vs. temperature profile (polynomial fitted curve illustrates the trend). Microscopic images of the standalone hydrogel at the annotated temperatures are shown as insets. **b** Reversible hydrogel (standalone) volume transition upon activation/deactivation of the microheater (polynomial fitted curve illustrates the trend). **c** A microfluidic valving characterization setup with a feedback-controlled pressure configuration. **d** The measured flow rate profile through a valve-gated microfluidic channel upon the periodic activation/deactivation of the valve. **e** Hydrogel layer fabrication procedure and layer-by-layer device integration scheme to realize microfluidic valving systems with different architectures. **f** Optical images of the representative fabricated hydrogel layers with different numbers/arrangements of hydrogels (a black substrate background is used to visualize the transparent hydrogel features). **g** Sequential optical images of progressive microfluidic routing and compartmentalization through illustrative serial, parallel, and tree microfluidic networks (constructed through integration with the same arrangement of hydrogels).

For robust on-body valving, the temperature at which the hydrogel’s volumetric transition occurs should be sufficiently above the skin temperature (~35 °C), such that the heat transfer from the skin to the valve does not result in significant hydrogel shrinkage and subsequent fluid leakage. By incorporating an ionizable monomer (MAPTAC) in the hydrogel structure^35^, the volumetric transition temperature of about 45 °C is achieved. As shown in Fig. 2a, the modified PNIPAM-based hydrogel exhibits about 40% shrinkage from its original size (based on the 2D imaged area) after ramping up its temperature above the LCST point. Reversibly, the hydrogel can recover back to its original volume, simply by deactivating the microheater (Fig. 2b). The observed asymmetry in the hydrogel shrinkage and recovery rates can be attributed to the difference between the outward and inward diffusion rates of the surrounding buffer solution that is leaving and entering the hydrogel, respectively^36^. Moreover, the corresponding shrinkage and recovery rates are found to be proportional to the hydrogel size as demonstrated in Supplementary Fig. 1b, c. In order to maintain a fast valve responsive time, we minimized the size of the hydrogel embedded inside the channel (circle-shaped with radius <1 mm). By setting up a pressure-controlled fluid flow configuration (Fig. 2c), the flow rate within a hydrogel-embedded and microheater-coupled microfluidic channel was monitored. As shown in Fig. 2d, upon deactivation/activation of the microheater, the flow rate within the channel correspondingly dropped to zero/recovered to its default value, illustrating the reversible, consistent, and periodic switching capabilities of the formed valve. The slower transient characteristic of the embedded hydrogel as compared to that of the standalone hydrogel (Fig. 2b vs.  2d) can be attributed to the surface contact forces acting on the embedded hydrogel. Furthermore, our device temperature characterization results show that operationally the valve opens at temperatures ≳45 °C (Supplementary Fig. 2).

To fabricate the valve interface in an array format and within a tape-based flexible microfluidic module, a simple and high-throughput fabrication and integration scheme is devised (Fig. 2e, Supplementary Figs. 3 and 4). The scheme involves fabricating the hydrogel array, microfluidic network structure, and electrode array on separate layers, followed by the vertical alignment and assembly of the layers^37^. We particularly positioned the microheater electrode array layer as the top layer (i.e., away from the skin, in which case intermediary layers serve as insulators), to minimize the heat conduction to skin. In our scheme, the hydrogel array and microfluidic network features are defined by a laser cutter, which can be programmed at a software level to rapidly render various arrangements and dimensions. The hydrogel arrays can be developed by simultaneously injecting PNIPAM precursor solutions into the respective defined features, followed by a one-step ultraviolet crosslinking procedure, altogether rendering the development process low-cost and highly scalable (Fig. 2f). Our vertical integration approach also allows the same arrangement of hydrogel arrays to form various microfluidic routing and compartmentalization networks, simply by integrating microfluidic layers with different architectures. For example, as shown in Fig. 2g, an arrangement of six hydrogels are used to gate microfluidic networks with serial, parallel, and tree-like architectures (for visualization purposes, a blue dye is embedded within the channels and the hydrogels are externally/locally heated).

### Active biofluid sampling from pressure-driven sources

**Fig. 3:**
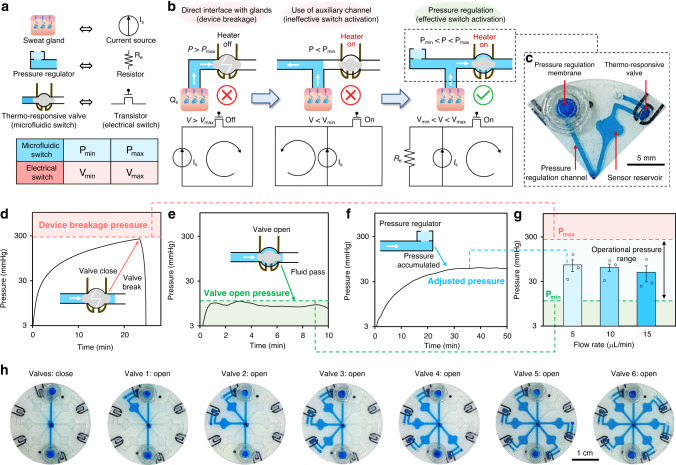
Elaboration, characterization, and demonstration of pressure-regulated valving. **a** An electric-hydraulic analogy. (*V*_min_: minimum turn-on voltage of the transistor switch; *V*_max_: maximum tolerable voltage of the transistor switch; *P*_min_: minimum required pressure to open the valve; *P*_max_: maximum tolerable pressure of the hydraulic valve). **b** Design rationale of the pressure regulation mechanism (assisted by the electrical circuit analogy). **c** Optical image of the implemented pressure-regulated valve. Real-time pressure recording for the characterization of the **d** maximum tolerable pressure, **e** minimum required pressure, and **f** regulated pressure. Input flow rate was set to 5 μL min^−1^. **g** Characterized accumulated pressure across pressure-regulated microfluidic channels at different flow rates. Error bars, mean ± s.e (*n* = 3 measurements from different devices). **h** Sequential optical images of progressive microfluidic routing and compartmentalization through an illustrative pressure-regulated six-compartment valving system (performed ex situ).

In order to adapt the demonstrated valving operation to actively sample, route, and compartmentalize epidermally retrievable biofluids from pressure-driven sources, pressure release mechanisms are necessary. Specifically, in the context of sweat as the target biofluid, a pressure release mechanism is devised to avoid excess pressure build-up from the sweat glands. Without such mechanism in place, valve breakage would occur, due to the high pressure caused by the accumulated sweat (as high as ~500 mmHg with an air-tight sealed interface)^38^. The problem at hand can be formulated with the aid of an electrical circuit-hydraulic analogy (Fig. 3a), involving a current source (delivering current level *I*_S_) and a transistor switch. Here, the minimum turn-on voltage for the transistor switch is denoted as *V*_min_ and its maximum tolerable voltage is denoted as *V*_max_ (corresponding to its breakdown voltage). When directly connecting the transistor (in its off mode) to the current source, the built-up high voltage difference across the transistor (*V*) inevitably leads to transistor breakdown (>*V*_max_). Similarly, as shown in Fig. 3b (left), when directly interfacing the air-tight closed valve (microfluidic transistor switch) with actively secreting sweat glands (with secretion rate *Q*_S_), the built-up high-pressure difference (*P*) across the valve inevitably leads to the valve breakage (*P* > *P*_max_, where *P*_max_ denotes the valve’s maximum tolerable pressure).

In both scenarios, the addition of a secondary parallel electric/hydraulic conductive path allows for redirecting the electrical current/fluid flow as a relief mechanism (Fig. 3b, center). However, the electric/hydraulic resistance of these paths must be tuned to ensure that the voltage/pressure across the respective switches is maintained above *V*_min_/*P*_min_ (where the switches are turned on). Electrically, this can be achieved by adding a parallel resistor (*R*_e_). Hydraulically, here, we use a membrane filter incorporated within an auxiliary microfluidic channel to render the desired hydraulic resistance, which effectively serves as a pressure regulation mechanism (Fig. 3b right, c).

To characterize *P*_max_ and *P*_min_ for our pressure-regulated valving interface, the same setup as that of Fig. 2c is used (with a programmed input flow rate of 5 μL min^−1^). As shown in Fig. 3d, the direct injection of fluid through the closed-valve microfluidic device (using a syringe pump) resulted in pressure built-up on the order of 300 mmHg (corresponding to *P*_max_), beyond which the device failed (due to leakage), as evident from the annotated drop in the measured pressure. Furthermore, the injection of fluid through the opened-valve microfluidic device resulted in ~10 mmHg pressure (corresponding to *P*_min_) across the device (Fig. 3e). Characterization of a microfluidic pathway, with the pressure regulation mechanism in place (Fig. 3f), illustrates the mechanism’s ability to effectively maintain the operational pressure (*P*) within the permissible pressure range (*P*_min_ < *P* < *P*_max_, Supplementary Fig. 5) for different input flow rates (Fig. 3g). In addition, Fig. 3h shows that a fully formed valving system (consisting of heater-coupled hydrogel valves and pressure regulating embodiments) can be successfully used to route and compartmentalize fluid in an addressable and electronically programmable manner.

### Flow rate-undistorted biomarker analysis

**Fig. 4:**
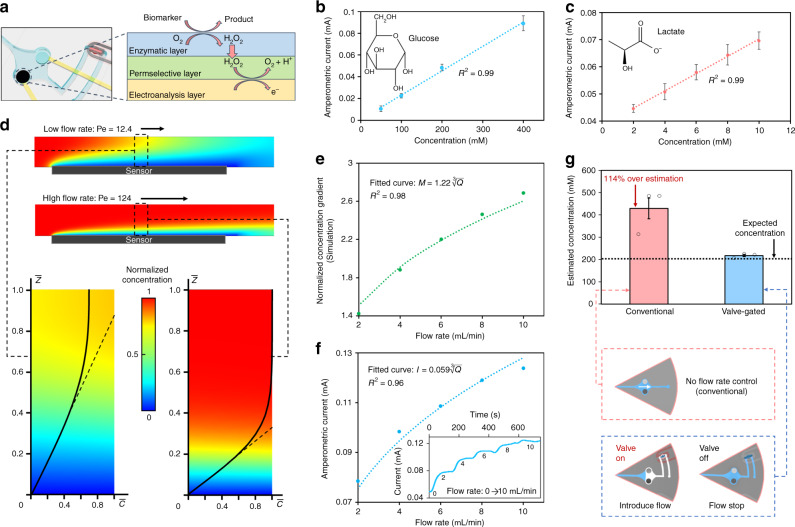
Demonstration of flow rate-undistorted biomarker analysis. **a** Reaction schematic of the developed sensor (embedded within a valve-gated compartment). Current response to target analytes for **b** a glucose sensor and **c** a lactate sensor. Error bars, mean ± s.e (*n* = 3 measurements from different sensors). **d** Simulated analyte concentration (gradient) profiles for relatively low and high flow rate conditions (low flow rate: *Q* = 1 µL min^−1^, resulting in Pe = 12.4, high flow rate: *Q* = 10 µL min^−1^, resulting in Pe = 124, assuming a channel transverse width of 2 mm and analyte diffusivity constant of 6.7 × 10^−6^ cm^2^ s^−1^). The annotated dashed lines tangent to the normalized concentration curves indicate the local analyte concentration gradient for the respective case. **e** Simulated local analyte concentration gradient at various flow rates (the values are normalized to that obtained for the case of 1 µL min^−1^). The curve fitted line indicates that simulated data points present a $\root {3} \of {Q}$ relationship. **f** Measured amperometric current response of a glucose sensor to 200 µM glucose solution introduced at various flow rates. The inset figure shows the corresponding measured real-time amperometric current response in the presence of progressively increasing flow rate (from 0 to 10 μL min^−1^). The curve fitted line indicates that simulated data points present a $\root {3} \of {Q}$ relationship. **g** Comparison of the estimated glucose concentration of a 200 µM glucose solution introduced at 5 µL min^−1^ (no valve) and 0 µL min^−1^ (corresponding to valve-gated condition). Error bars, mean ± s.e (*n* = 3 measurements from different sensors).

To demonstrate the utility of the devised active biofluid management system, biochemical sensing interfaces are developed and incorporated in the sensing chamber of the valve-gated compartments (upstream of each compartment channel as shown in Fig. 4a), following the previously reported mediator-free enzymatic sensor development methodology^39^. We specifically adapted the sensing interfaces to target glucose and lactate as examples of informative metabolites. As illustrated in Fig. 4a, the corresponding sensing interfaces comprised of: (1) an enzymatic layer (glucose oxidase or lactate oxidase) to catalyze the oxidation of target molecules and generate hydrogen peroxide (H_2_O_2_) as a detectable byproduct; (2) a permselective membrane (poly-*m*-phenylenediamine) to reject interfering electroactive species; and (3) an electroanalysis layer (platinum) to detect the generated H_2_O_2_. The response of the glucose and lactate sensors were validated within the respective analytes’ physiologically relevant concentration range in sweat^40,41^. As shown in Fig. 4b, c, for both sensors, linear relationships were observed between the measured current responses and target analytes’ concentration levels (*R*^2^ = 0.99, for both sensors).

The active biofluid flow control achieved by the valving system can be leveraged to address sensor-level challenges relevant to wearable biomarker sensing. In particular, here, the valving capability was utilized to decouple the confounding influence of flow rate variability on sensor response, an issue which is well-reported in the context of conventional lab-on-a-chip platforms^29–32^, but overlooked by previously reported wearable sensors.

In a generalizable continuous microfluidic electrochemical sensing setting, the response of the sensor is flow rate-dependent, because of the central role of advective flow in transporting analytes to the sensor^42^. In the case of electrochemical sensing, the sensor current response (*I*) is proportional to the flux of analyte molecules onto the sensor surface, which in turn is directly proportional to the local concentration gradient (*M* = $\frac{{\partial c}}{{\partial z}}$). In that regard, determining the local concentration gradient requires the consideration of various coupled phenomena, including advective and diffusive analyte transport to the sensor surface, and the reaction rate at the sensor surface. As described in the Supplementary Note, the coupled problem at hand can be simplified by assuming the sensor has a high surface reaction rate, and that advection is the dominant form of analyte transport (manifested as Peclet number ≫ 1, due to the relatively high sweat rate *Q* ~ 1–10 µL min^−1^ during active secretion). The theoretical analysis based on these assumptions leads to the $I \propto M \propto \root {3} \of {Q}$ relationship.

This relationship was validated through finite element analysis (FEA) (COMSOL), where we simulated the analyte concentration profile at the sensor surface in response to various continuous flow rates (within the physiologically relevant range of sweat secretion rate). As shown in Fig. 4d, e, the concentration gradient on the sensor surface increased along with the flow rate in the microfluidic chamber, in which *M* was proportional to $\root {3} \of {Q}$ (*R*^2^ = 0.98). Similarly, the measured amperometric current of a representative glucose sensor presented a cube-root relationship with *Q* (Fig. 4f, *R*^2^ = 0.96), which is in agreement with our theoretical analysis.

Practically, without accommodating for the influence of dynamically varying flow rate (during on-body measurements), if conventional calibration methods are followed (which are performed at zero flow rate, ex situ), inaccurate biomarker measurements will inevitably be obtained. This problem can be resolved by leveraging the devised valving mechanism, as it allows for performing analysis in a sample-and-hold manner. To elaborate, in a valve-gated sensing chamber, the valve can be opened, to allow for the introduction of the sample into the sensing chamber, and closed, to allow for sample compartmentalization and sensing at zero flow rate, thus effectively decoupling the confounding influence of flow rate variability. To demonstrate the influence of flow rate variability, the response of a representative glucose sensor to an introduced sample (containing 200 µM glucose) was monitored at 5 µL min^−1^ (no valve) and 0 µL min^−1^ (corresponding to valve-gated condition), and the corresponding estimated concentrations were derived by referring to the calibration curve (obtained at 0 µL min^−1^). As shown in Fig. 4g, the conventional setup overestimated the glucose concentration by 114%, whereas the valve-gated condition accurately estimated the glucose concentration.

### Contextually relevant on-body biomarker analysis

**Fig. 5:**
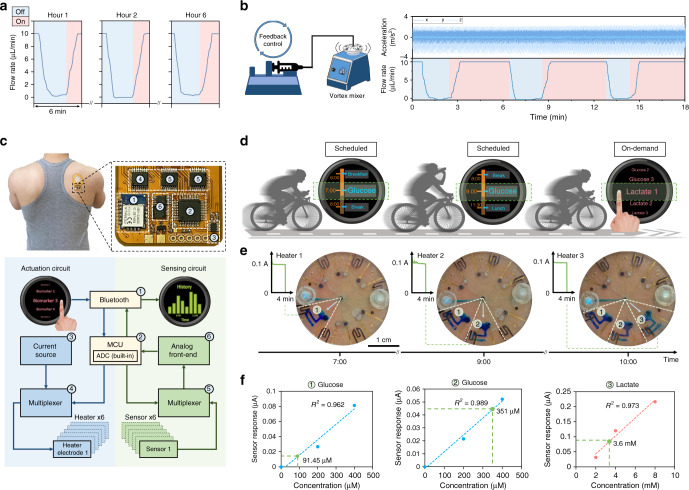
Integration and characterization for contextually relevant on-body biomarker analysis. **a** Ex situ characterization of the prolonged operation of the pressure-regulated valve (performed over 6 h). **b** Ex situ characterization of the high-fidelity operation of the pressure-regulated valve in the presence of vortical vibration. The vibrational acceleration profiles are presented in the top half, and the characterized flow rate profile is captured in the bottom half. **c** Optical image of a representative fully integrated programmable epidermal microfluidic valving system applied on the back of a subject with a zoomed-in view of the FPCB electronic components. The block diagram details the circuit-level valve actuation and signal-processing operations. **d** Illustration of the planned study for scheduled/on-demand sweat sampling during physical activity (cycling). **e** Optical images of intermittently sampled, routed, and compartmentalized sweat on-body (visualized with the aid of blue dyes, embedded within the compartments). Three valves were sequentially activated/deactivated at programmed timepoints during a physical exercise. The inset figures show the characterized electrical current through the respective valves’ microheaters (activated for 4 min). **f** Measured sweat glucose and lactate concentrations based on-body sensor readouts (green data points). The corresponding calibration curves (dashed lines) were constructed by linear fitting the measured sensor responses to three reference samples with known analyte concentrations (blue/red data points in the case of glucose/lactate sensors). Sweat glucose readouts were obtained by the valve-gated sensing compartments 1 and 2, before and after a scheduled beverage intake event, respectively. The sweat lactate readout was obtained by the valve-gated sensing compartment 3 upon on-demand user activation.

In order to apply the devised pressure-regulated valving system for on-body biofluid management and biomarker analysis, we first evaluated the system’s operational stability during prolonged use and in the presence of motion artifacts. In that regard, we applied the flow rate characterization setup (same as that used in Fig. 2c) to quantitatively monitor the performance of a pressure-regulated valve in an ex situ setting. First, to assess its stability during a prolonged testing period, we sequentially activated and deactivated the valve at set timepoints over a period of 6 h. Figure 5a shows the flow rate, injected by the pressure-driven syringe pump, was successfully reduced to zero and back to its default value upon deactivation and activation of the valve, respectively. In addition, Supplementary Fig. 6 illustrates that for our context, hydrogel dehydration does not affect the intended valving functionality, as evident from the maintenance of a relatively constant pressure—across a valve-gated channel—over an extended amount of time (8 h). The minimal impact of hydrogel dehydration can be attributed to the small size of the outlets, minimizing the evaporation rate. Furthermore, to evaluate the stability of the valving system against motion artifacts, its performance was characterized under oscillatory motion (amplitude: ~3 m s^−2^ at 5 Hz^43^, generated by a vortex mixer). The measured flow rate profile, shown in Fig. 5b, indicates the successful opening and closing of the valve. Further ex situ and in situ characterization results, shown in Supplementary Figs. 7 and 8, provide insight into the robustness of the valving interface in the presence of mechanical deformation and unconstrained body motion. Altogether, these characterization results illustrate the preserved functionality of the valve over the test periods/conditions, informing the robustness of the valving operation for on-body application.

To realize a wearable valve-enabled bioanalytical platform with seamless control command and biomarker data communication capabilities, the sensor array-coupled valving system is interfaced with a custom-developed wireless FPCB (Supplementary Figs. 9 and 10). Structurally, the FPCB module is 100-μm thick, and its base material is polyimide, the Young’s modulus of which is on the same order as those of the materials used in the microfluidic module’s structure (Supplementary Table 1). In case a higher degree of mechanical flexibility is needed (e.g., when interfacing high curvature areas), other base materials with lower Young’s modulus can be used to construct the circuit board^44^. Figure 5c illustrates the operational block diagram of the FPCB, which is capable of rendering multichannel valve actuation and signal processing. Depending on the context at hand and the desired mode of analysis, an activation signal for the designated valve-gated sensing compartment is transmitted to the FPCB’s microcontroller unit (MCU). This activation signal can be generated through a scheduled timetable or on-demand (initiated by the user). Upon processing the received command, and with the aid of a multiplexer unit, the MCU selects the appropriate actuation channel to power the corresponding microheater by a current source, subsequently opening the desired valve. Subsequently, the harvested biofluid is routed to the selected compartment. Then, following MCU-generated instructions, the valve closes, and the sensor response is recorded and processed by an analog front-end (consisting of potentiostat and low-pass filter units) via the multiplexer-selected sensing channel. The signal processed by the analog front-end is then translated to digital at the MCU level, and wirelessly communicated to a user interface. The user interface can be used to display the acquired biomarker information in real-time and to store it in the user’s database.

The devised wearable valve-enabled bioanalytical platform was deployed for sweat sampling at scheduled and on-demand timepoints, to illustrate the platform’s capability for contextually relevant biomarker analysis applications (Fig. 5d). Accordingly, the platform was mounted on the back of a subject engaged in cycling (with the aid of a skin-adhesive layer, which provides adequate adhesion force to maintain the platform on the skin, Supplementary Fig. 11). Prior to on-body deployment, we activated the microheaters and monitored the electrical current passing through them to verify their operation. As can be seen from the on-body experiment, shown in Fig. 5e, the secreted sweat, at set scheduled/on-demand timepoints, was routed to and compartmentalized within the desired compartments (following a 4-min microheater activation time-window), while other compartments were protected. This time-stamped biofluid acquisition capability can be exploited to take contextual biomarker readings. As shown in Fig. 5f, the platform was programmed to take glucose readings before and after a scheduled beverage intake (Trutol, containing 50 g per 296 mL of dextrose) event, and sweat lactate level was measured on-demand as per the user’s command. Specifically, the biomarker readouts indicated the subject’s sweat glucose level was elevated after glucose intake, and the measured sweat lactate level was within an expected range (in agreement with previously reported studies)^45,46^. Supplementary Fig. 12 further validates our solution’s suitability for compartmentalization and sensor operations on-body. Our human subject experiment results, captured by the final device (prior to COVID-19 pandemic), were limited to the ones presented in this paper—no further human subject experiments were performed due to safety concerns around COVID-19. To provide physiologically meaningful interpretations of such sensor readouts, future large-scale studies should be conducted, aiming to contextualize the measured sweat biomarker concentrations in relation to relevant inter/intra-individual physiological variabilities (e.g., gender, muscle density, and body hydration).

## Discussion

By devising a programmable epidermal microfluidic valving system, we achieved in situ active biofluid management, which is central to the realization of autonomous and advanced biofluid processing and analysis capabilities underpinning the envisioned lab-on-body platforms. The core of the microfluidic system is a network of individually addressable microheater-controlled thermo-responsive hydrogel valves, fabricated following a high-throughput, low-cost, and scalable fabrication scheme. A devised electronic-hydraulic analogy provided the basis for developing a pressure regulation mechanism (integrated within the microfluidic valving system), which was used to harvest biofluid, in situ, from pressure-driven bio-interfaces (here, sweat glands). While here, we demonstrated wearable valving in the context of exercise-induced sweat sample compartmentalization, the presented technology can be equivalently adopted for the compartmentalization of iontophoretically induced sweat (where the secretion rate is on the same order as that of the exercise-induced sweat^47,48^). In that regard, a dedicated programmable iontophoresis interface should be integrated, which enables contextually relevant sweat sampling in sedentary subjects^2^.

The active fluid control achieved by this system is harnessed to create new wearable bioanalytical capabilities at both the sensor and system levels. At the sensor level, the valving capability is exploited to decouple the previously overlooked issue (in wearable biosensing) of flow rate influence on sensor response. Accordingly, first, a mass transport-centered theoretical model was formulated and presented within the framework of wearable microfluidic sensing, and subsequently, validated by simulation and experimental results. Then, to decouple the influence of flow rate, we exploited the valving capability to perform analysis in a sample-and-hold manner, allowing for obtaining undistorted biomarker readings. At the system level, addressable biofluid routing and compartmentalization achieved by valving were leveraged to implement programmable sensor selection/protection. Through integration with an FPCB and seamless bilateral communication with consumer electronics, the valving system was adapted for on-body biomarker analysis, where the demonstrated capabilities converged to render contextually relevant (scheduled/on-demand) biomarker data acquisition.

The demonstrated technology can be equivalently adapted to implement sample processing operations such as incubation, reagent delivery, and purification, thus enabling the realization of advanced assays (particularly, those that have already been demonstrated in lab-on-a-chip settings) to create new biomarker detection solutions in a wearable format. The valve-enabled sample processing and analysis operations can be positioned as addressable compartments to form the building blocks of multistep and multichamber bioanalytical functions within microfluidic architectures, allowing for the execution of synchronous/asynchronous sequential and parallel bioanalytical objectives autonomously. On a broader level, the convergence of the active biofluid management capabilities achieved by the presented technology and other active actuation modalities allows for the creation of fully autonomous lab-on-body platforms to monitor the biomarker profiles of individuals at the point-of-person, thus informing personalized and actionable feedback toward improving the individual’s health.

## Methods

### Fabrication procedure

The wearable valve-enabled bioanalytical platform is composed of multiple vertically stacked layers, which can be listed from the bottom to the top as: (a) a double-sided skin-adhesive film, (b) a biochemical sensing electrode array patterned on a polyethylene terephthalate (PET, ~100 µm, MG Chemicals) substrate, (c) a microfluidic layer for sweat sampling, routing, and compartmentalization, (d) a thermo-responsive hydrogel array layer, (e) a microheater electrode array for valve switching, and (f) pressure regulator embodiments. These components are fabricated following the ensuing described protocols.

The microfluidic module is constructed by vertical assembly of double-sided tapes (170-µm thick, 9474LE 300LSE, 3 M) and transparent PET film layers. Microfluidic features such as microchannels and VIAs (Vertical Interconnect Access) were fabricated by laser-cutting (VLS2.30, Universal Laser Systems). Through the vertical alignment of the microchannels and VIAs, fluidic connections were made between different layers of the microfluidic module, rendering a 3D microfluidic structure.

The microheater electrode array was patterned on PET by photolithography using a positive photoresist (MicroChemicals AZ5214E), followed by the evaporation of 20 nm Cr, 100 nm Au, and 20 nm Ti. The sensor electrode array was also patterned on PET by photolithography using positive photoresist (MicroChemicals AZ5214E), followed by the evaporation of 20 nm Cr and 100 nm Au. The lift-off step was performed in acetone. To establish seamless electrical connections, in a spatially efficient manner between the microheater/sensor array layers and the FPCB, double-sided adhesive anisotropic conductive films (ACFs, 9703, 3 M, 50 µm) were used as VIAs to connect the contact pads of the board (located on its front- and back-sides) to the layers. Specifically, for the microheater electrode array, the connections were made to the front-side of the FPCB (from the top), and for the sensor electrode array, the connections were made to the back-side of the FPCB (from the bottom).

The thermo-responsive hydrogels were prepared by mixing 0.545 g NIPAM (Sigma-Aldrich), 0.0297 g *N*,*N*′-methylenebisacryl-amide (Sigma-Aldrich), 0.75 mL dimethyl sulphoxide (Sigma-Aldrich), 0.25 mL deionized water, 0.02 mL [3-(methacryloylamino)propyl]trimethylammonium chloride (MAPTAC, Sigma-Aldrich) solution (50 wt.% in water), and 0.0385 g 2,2-dimethoxy-2-phenylacetophenone (Sigma-Aldrich). This mixture was then sonicated in a water bath for 30 min at 48 °C with a sonication frequency of 40 kHz. Next, the mixture was injected and cast into custom-designed tape-based molds (laser-cut with the desired features), followed by a photo-polymerization step (405 nm ultraviolet light, Formlabs Form Cure, intensity: 1.25 mW cm^−2^ and exposure time: 2 min). The crosslinked hydrogels were immersed in a DI water bath for at least 12 h, prior to their deployment for the planned characterization/validation experiments.

The pressure regulators were constructed by embedding laser-cut filter membranes (GD 120 Glass Fiber Filter, Advantec MFS Inc.) in between two double-sided tape layers (170-µm thick, 9474LE 300LSE, 3 M), forming a sandwiched structure. Epoxy (Devcon) was used to seal the gap between the layers.

To develop biochemical sensing interfaces, first, platinum-based working electrodes were constructed by electrochemically depositing (−0.1 V vs. Ag/AgCl, 600 s) a platinum nanoparticle (PtNP) layer onto the designated sensor electrodes (Au-based) using an aqueous solution containing 2.5 mM Chloroplatinic acid (H_2_PtCl_6_·6H_2_O, Sigma-Aldrich) and 1.5 mM formic acid (Sigma-Aldrich). Next, a poly-*m*-phenylenediamine (PPD) layer was electrochemically deposited onto the PtNP/Au electrode (0.85 V vs. Ag/AgCl, 300 s) in a phosphate-buffered saline (PBS) solution (pH 7.2; Gibco PBS, Thermo Fisher Scientific) containing 5 mM *m*-phenylenediamine (Sigma-Aldrich). The constructed PPD/PtNP/Au electrode was then washed (with DI water) and dried at room temperature. Reference electrodes were constructed by drop-casting Ag/AgCl ink onto the designated electrodes (Au-based). Then, the deposited layer was dried at 70 °C for 30 min. It is worth noting that the Ag/AgCl reference electrode construction took place in between the PtNP and PPD deposition steps (when constructing the working electrode). Supplementary Table 2 summarizes the chemical composition of the enzymatic sensing interfaces.

To develop the glucose sensor, 0.3 µL of a 1:1 (v/v) mixture of 1% chitosan solution and glucose oxidase (50 mg ml^−1^ in PBS, pH 7.2; Sigma-Aldrich) was coated onto the PPD/PtNP/Au electrode (1.13 mm^2^). The 1% chitosan solution was prepared by dissolving chitosan (Sigma-Aldrich) in a 2% acetic acid (Sigma-Aldrich) solution at 60 °C for 30 min. To develop the lactate sensor, a 0.3 µL of 1:1 (v/v) mixture of bovine serum albumin (BSA, Sigma-Aldrich) stabilizer solution and lactate oxidase solution (50 mg ml^−1^ in PBS, pH 7.2; Toyobo) was coated onto the PPD/PtNP/Au electrode (1.13 mm^2^) and dried at room temperature for 1 h. The BSA stabilizer solution was prepared by adding 0.8% (v/v) of 25 wt.% glutaraldehyde solution (GAH, Sigma-Aldrich) in a PBS solution containing 10 mg ml^−1^ BSA. Then 0.3 µL of PVC solution (0.375 wt.% in Tetrahydrofuran; Sigma-Aldrich) was deposited twice (separated by 1 h) onto the electrode surface to form a lactate diffusion limiting layer. All sensors were allowed to dry overnight at 4 °C while being protected from light, prior to their deployment for the planned characterization/validation experiments.

### Characterization of hydrogel’s thermo-responsivity

To characterize the effect of temperature on hydrogel shrinkage, a circular hydrogel was placed on top of a hot plate (Isotemp, Fisher Scientific). The temperature of the hot plate was gradually increased, with 2 °C temperature increments and 2 min of wait time (allowing the hydrogel to reach steady-state). In order to characterize the fabricated microheater-coupled hydrogel’s reversible response, a DC power supply (Keithley 2230-30-1, Keithley Instruments Inc.) was used to apply 2.8 V across the microheater electrodes. This configuration allowed for immediate delivery and removal of heat, and the characterization of the hydrogel’s transient volumetric transition. Optical imaging was performed, followed by image analysis, to quantify the changes in the area of the hydrogel. The output datasets were plotted with Excel (version 2007, Microsoft) both for this and other experiments.

### Flow control characterization of the valve-gated channel

To characterize the flow control capability of the hydrogel valve, the inlet of a valve-gated microfluidic channel was connected to a proportional–integral–derivative (PID) controlled syringe pump (PHD ULTRA^TM^ CP, Harvard Apparatus), which was configured to maintain a pressure of 15 mmHg with a flow rate range of 0–10 µL min^−1^. To control the syringe pump, via a feedback loop, the device inlet pressure was measured and transduced with the aid of a pressure sensor (Blood Pressure Transducers, APT 300, Harvard Apparatus) and a transducer amplifier module (TAM-D, Harvard Apparatus). The flow rate data during the periodic valve activation/deactivation was recorded by PID Pump Data Log software (Harvard Apparatus) and processed by applying a Savitzky–Golay filter to remove the measurement artefacts (e.g., pump’s mechanical noise). Furthermore, we optically verified that near-zero readings (processed PID system data) correspond to a zero flow rate when the valve was closed. The output datasets were processed with Origin (version 2018, OriginLab Corporation) both for these experiments.

### Pressure characterization

Three microfluidic device configurations were used to correspondingly characterize the device breakage pressure, valve open pressure, and adjusted pressure by the regulator: (1) a microfluidic channel with a closed embedded valve; (2) a microfluidic channel with an open embedded valve; and (3) a microfluidic channel with an auxiliary pressure regulator channel. In separate experiments, each configuration was connected to a syringe pump, which was programmed to inject a solution at the constant flow rate of 5 µL min^−1^ into the test device’s channel. Specifically, in order to characterize the valve’s maximum tolerable pressure (*P*_max_), where the first device configuration was used, the solution was continuously injected until the device breakage occurred (evident from a drop in the measured pressure). The corresponding pressures across the inlet and outlet of the channels of the test devices were measured by a pressure sensor (Blood Pressure Transducers) and recorded by the PID Pump Data log software (Harvard Apparatus).

### Ex situ characterization of valving fidelity

To assess the operational fidelity of the devised valving system, six-compartment pressure-regulated microfluidic valving devices (Fig. 3h) were tested for stability under induced motion artifacts and prolonged use. For both cases, the devices’ inlets were connected to the aforementioned flow control characterization setup, which allowed for continuous solution injection and device flow rate monitoring. During the valve activation/deactivation, the flow rate data were recorded, and subsequently post-processed with the aid of the PID Pump Data Log software and filters. To test for prolonged valving operation, a designated compartment was sequentially activated and deactivated at set timepoints over a period of 6 h. For the motion artifacts test, an accelerometer (on a smartphone) was affixed to the device and a vortex mixer (Fisher Scientific), which was adjusted to mimic 3D oscillatory acceleration conditions (amplitude: ~3 m s^−2^ at 5 Hz generated by a vortex mixer).

### Biosensor response characterization

To characterize the developed enzymatic sensing interfaces, amperometric measurements were performed at +0.5 V vs. Ag/AgCl in the sample solution (e.g., glucose and lactate) with a potentiostat (CHI 660E, CH Instruments). Calibration plots of glucose and lactate sensors were obtained by recording the amperometric responses in a series of PBS solution containing different concentrations of the target analytes (d-(+)-Glucose: from 50 to 400 µM, Sodium L-lactate: from 2 to 10 mM, Sigma-Aldrich). To investigate the flow rate effect on the sensor performance, amperometric responses were recorded while continuously injecting the PBS solution containing 200 µM glucose into the glucose sensing chamber with the flow rate incrementally ramped up from 2 to 10 µL min^−1^ (controlled by a syringe pump, Harvard Apparatus).

### FEA of the flow rate influence

FEA software, COMSOL 5.2, was used to simulate the concentration profile of a model analyte inside a microfluidic channel under various laminar flow rate conditions. In the simulation software, two simulation packages, “laminar flow” and “transport of diluted species”, were employed and coupled in the context of a 2D microfluidic channel. The channel was set to be 170 µm in height, which is the same as the experimental setup. The sensor (1 mm in length) was positioned far enough from the inlet, allowing for the establishment of a pressure-driven Poiseuille flow profile. Input average flow velocities were determined in relation to the experimentally relevant volumetric flow rate (1–10 µL min^−1^) and by assuming a channel height of 170 µm and width of 2 mm. The range of the volumetric flow rate is selected based on the previously reported active sweat secretion rates^47,48^ and our device sweat collection area (8 cm^2^). The analyte bulk concentration at the inlet of microfluidic channel (*c*_0_) was set to 200 µM and the concentration at the sensor surface was set to zero (following the high surface reaction rate assumption). The diffusion coefficient of target analyte (here, glucose) was set as 6.7 × 10^−6^ cm^2^ s^−1^. The concentration gradient of the analyte at the vicinity of the sensor surface (at its midpoint) was extracted to infer the analyte flux onto the sensor.

### FEA of the strain distribution

COMSOL 5.2 was used to simulate the mechanical behavior of the developed microfluidic valve device under bending conditions. A representative 2D model of a microfluidic valve (cross-view) was used for the mechanical analysis, in which case it was assumed no delamination between layers/components being considered. Bending force was applied on the bottom PET layer with the vertical displacement of the two corners set to zero. The magnitude of the force was adjusted based on the simulated bending curvature (matching the experimental results). The modeled device geometry and mechanical properties were set based on those of the fabricated device.

### Wireless system operation and interface

Wireless addressable valving and biomarker analysis was realized with a custom-developed FPCB. An on-board MCU (ATmega328, Microchip Technology), with the aid of analog multiplexers (MAX4781, Maxim Integrated, USA), was utilized to select the desired channels for valve actuation (via activating the microheater) and signal acquisition from the corresponding sensors. The selection of the valves results in the electrical connection of the designated microheater contact pads with a programmable current source (LT3092, Linear Technology, USA). The selection of the sensing channels results in the electrical connection of the designated sensing electrodes’ contact pads with a potentiostat chip (LMP91000, Texas Instruments). The potentiostat chip was programmed to apply 0.5 V across the working and the reference electrodes, and to convert the acquired sensor current signal to voltage through the internal transimpedance amplifier. The processed signal by the potentiostat was then filtered by a fifth-order low-pass filter (MAX7422, Maxim Integrated, USA) with a cutoff frequency of 1 Hz and translated into the digital domain with the aid of the MCU’s built-in 10-bit analog-to-digital converter. By interfacing the MCU with a Bluetooth module (AMB2621, Wurth Elektronik, Germany), wireless, bilateral, and real-time communication of user commands and sensor output data with Bluetooth-enabled consumer electronics was achieved (e.g., smartphone or smartwatch).

An Android-based application was developed for a Moto 360 (2nd generation) smartwatch (Motorola, Inc.) to implement a user-friendly interface for programmable biomarker acquisition timepoints (scheduled or on-demand). The application is primarily developed with the aid of the Android software development kit (Android Studio package) to implement key functionalities such as data communication, display, storage, and interactive user interface. The smartwatch application features three main functions, namely: History, Scheduled, and On-demand. These functions are accessible through a main selection screen that also displays the current time. The History function stores (in a local buffer) and displays the most recently recorded biomarker data in the format of a time series bar chart (based on the data stream received from the FPCB module, via Bluetooth). The Scheduled function displays the defined schedule for biomarker recording. This function can also transmit the sensor selection activation command (an integer index between 1 and 6) to the FPCB module (via Bluetooth)—in accordance to the defined schedule. The On-demand function overrides the schedule and to transmit the sensor selection activation command on-demand. This function features a scrolling list from which the user can select the desired sensing compartment. An intermediary smartphone, preloaded with a programmed Android service, can be used to mediate the smartwatch and FPCB communications for data storage.

### Power delivery

The custom-developed wireless FPCB was powered by a single rechargeable lithium-ion polymer battery with a nominal supply voltage of 3.7 V (Supplementary Fig. 13). This FPCB features a power management module that utilizes a voltage regulator chip (AP7313-33SAG-7, Diodes Incorporated, USA) to provide a stable voltage level of 3.3 V, to power up the rest of the circuit modules (Supplementary Figs. 9 and 10). The system draws on an average of 9 mA from the battery when no heater is on and 109 mA when activating a heater. Ultimately, the choice for the battery’s capacity/discharge current rating depends on the intended modes, duration, and frequency of operations. In the conducted exemplary human subject study (Fig. 5), where the trial took 3 h, and sweat sampling and analysis where performed at three timepoints (each valve activation over 4 min), a battery with a capacity rating on the order of 48.8 mAh (=109 mA × 0.2 h + 9 mA × 3 h) and discharge capability on the order of 100 mA was needed—the requirements that can be met by the lithium-ion polymer batteries widely used in commercialized wearable technologies (e.g., smartwatches).

### On-body validation of the microfluidic valving system

The flexibility and the adhesive surface of the constructed devices allowed for their placement on various body parts. To validate sweat sampling, routing, compartmentalization, and analysis, the developed devices were mounted onto the back of a healthy adult male volunteer engaged in cycling sessions. Prior to on-body application, the microheaters’ operations were verified by monitoring the current passage through the designated microheater electrodes. Furthermore, by incorporating thermocouple wires at the device/skin interface we validated that the effect of microheater activation on skin temperature is minimal (<4 °C). In addition, the sensors were pre-calibrated following the same procedure described in the “Biosensor response characterization” section. To visualize sweat sampling, blue dyes (FD&C Blue) were embedded within the constructed compartments. For on-body sweat glucose analysis, the subject was scheduled and instructed to consume a high-glucose beverage (Trutol, containing 50 g per 296 ml of dextrose) in between two exercise sessions. Here, the device was programmed to activate the glucose analysis-designated valves before and after beverage intake. For on-body sweat lactate analysis, the subject manually activated the corresponding valve at an unscheduled timepoint (representing on-demand device operation). For each analysis, sweat sampling was performed over a period of four minutes (after activating the valve), and biomarker analysis was performed for 100 s when the valve was turned off.

### IRB approval for human subject testing

The conducted human subject experiments were performed in compliance with the protocols approved by the Institutional Review Board (IRB) at the University of California, Los Angeles (IRB no. 17-000170). All subjects gave written informed consent before participation in the study.

### Reporting summary

Further information on research design is available in the Nature Research Reporting Summary linked to this article.

## Supplementary information

Supplementary Information

Reporting Summary

## Data Availability

All data needed to evaluate the findings and conclusions in the paper are present in the paper and/or Supplementary Information and can be accessed through the Open Science Framework at https://osf.io/dqmgc/?view_only=262e07c22c56492380229dce9f0543c2 (10.17605/OSF.IO/DQMGC).
