# Supplementary material for: A programmable epidermal microfluidic valving system for wearable biofluid management and contextual biomarker analysis
Source: Nat Commun. 2020 Sep 2;11:4405. doi: 10.1038/s41467-020-18238-6 (PMC7467936; doi:10.1038/s41467-020-18238-6)
Supplement: Supplementary file 1 — Supplementary Information [file 41467_2020_18238_MOESM1_ESM.pdf]

Supplementary Information for

A Programmable Epidermal Microfluidic Valving System for Wearable Biofluid Management and Contextual Biomarker Analysis

Lin et al.

Supplementary Note

Theoretical analysis of the flow rate influence on the electrochemical sensor response.

In the general case of modeling the response of a microfluidic electrochemical sensing system, analyte transport (by advection and diffusion) and surface reaction must be simultaneously considered. However, in the context at hand, because of the high enzymatic catalytic activity (i.e., high surface reaction rate), it can be assumed that the response of the electroenzymatic sensor is completely controlled by analyte transport onto the sensor surface[1]. Accordingly, the enzymatic current response can be presented as

$$I = nFAJ \quad (1)$$

, where *n* is the number of electrons in the electro enzymatic reaction, *F* is Faraday's constant, *A* is the sensing electrode area, and *J* is the analyte flux (molecules per area per time) onto the sensor surface.

When no flow rate is present, the analyte consumption on the sensor surface creates a growing analyte depletion zone with a thickness of $\delta \propto \sqrt{Dt}$, where *D* is the diffusion coefficient of the target analyte and *t* is time. Accordingly, the analyte molecules diffuse along the concentration gradient, resulting in analyte flux *J* onto the sensor surface, where $J = D\nabla c$. As the first-order approximation, the gradient ∇c can be simply equated to the difference in the analyte concentration in bulk (c_0) *vs*. immediate vicinity of the sensor surface (c_s, where $c_s \approx 0$, due to the assumption of relatively high surface reaction rate) divided by the depletion thickness (δ), hence:

$$J \approx \frac{D(c_0 - c_s)}{\delta} \approx \frac{Dc_0}{\delta} \quad (2)$$

Despite the continuous growth of the depletion thickness with time, if one takes measurements at a fixed timepoint, the proportionality of *J* in relation to c_0 can be exploited to establish a linear calibration curve (current response *vs.* analyte concentration, *e.g.*, Fig. 4b,c).

When performing sensing in the presence of advective flow (with volume flow rate *Q*) inside a microfluidic chamber, because of the continuous supply of analytes, the advection halts the growth of the depletion zone, setting a steady-state δ_s. For the case where the advection transport is stronger than analyte diffusion (captured by the non-dimensional Peclet number, $Pe = \frac{Q}{DW} \gg 1$), the advective delivery of analytes results in the compression of the depletion zone following the relationship below:

$$\frac{\delta_S}{L} \sim \sqrt[3]{\frac{DH^2W}{QL^2}} \quad (3)$$

, where L is the length of the sensor and W and H are the chamber width and height, respectively[2]. Combining equations (1-3) yields $I \propto J \propto \sqrt[3]{Q}$, a trend which is in agreement with both the simulation and experimental results (Fig. 4e,f).

Supplementary Figures

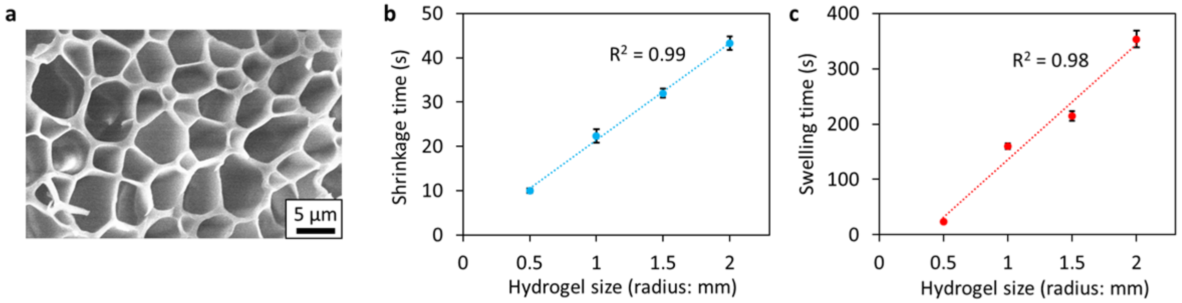

Supplementary Figure 1: a An illustrative Scanning Electron Microscopy (SEM) of a freeze-dried hydrogel with 4% BIS (imaged once). The features are consistent with previously reported observations[3]. **b-c** Characterization of the hydrogel volume transition time *vs* hydrogel size upon activation/deactivation of the microheater for shrinkage (**b**) and swelling (**c**). Shrinkage and swelling times are defined as the time it takes for the hydrogel shrinkage/expansion to settle within 1% of its steady-state volume upon (activation/deactivation of the microheater). Error bars, mean ± s.e (n = 3 measurements from different hydrogels).

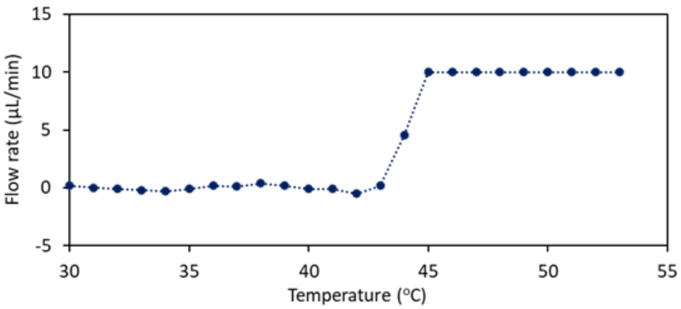

Supplementary Figure 2: Flow rate *vs*. hydrogel valve temperature profile (pressure is set as 15 mmHg). The valve is opened when the temperature exceeds 44 ºC. Y-axis indicates the averaged continuous recordings of the flow rate for each temperature condition.

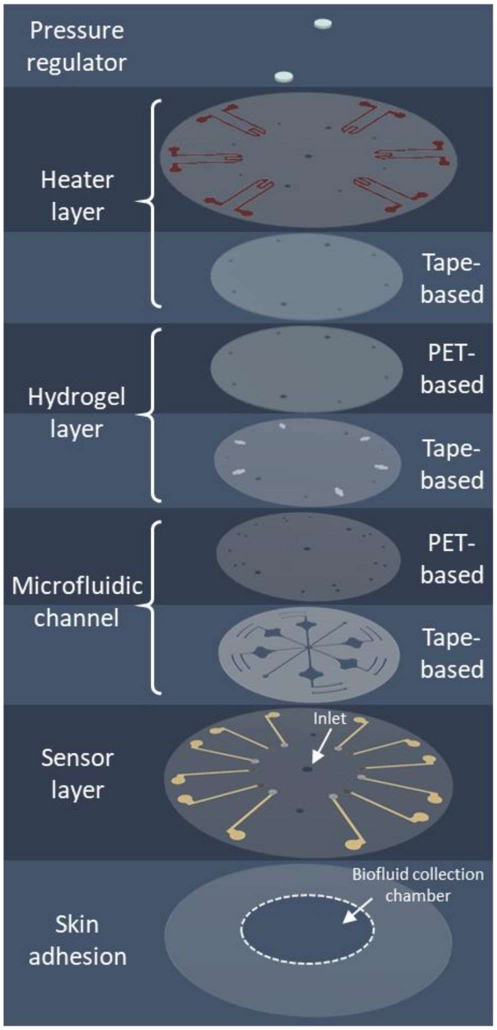

Supplementary Figure 3: Exploded view of the epidermal microfluidic valving system, which is constructed by the vertical integration of pressure regulator/hydrogel embodiments, laser-cut microfluidic channel layers, microheater/sensor array layers, and a double-sided adhesive skin adhesion layer.

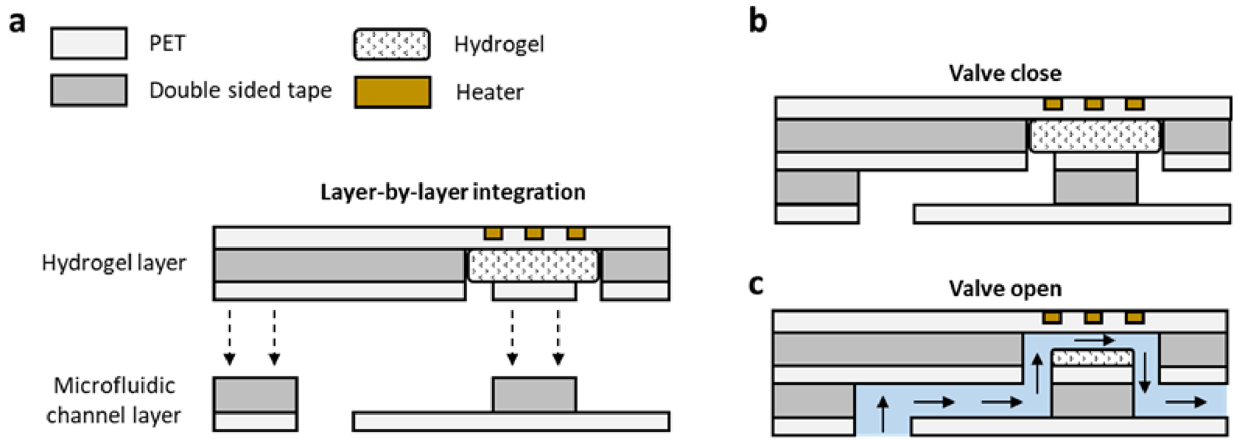

Supplementary Figure 4: a Demonstration of the layer-by-layer integration method to form the valve interface. **b,c** Illustrations of (b) valve closure when the microheater is off, and (c) valve opening when the microheater is on. Microheater activation causes hydrogel shrinkage, allowing incoming biofluid to travel through the channel.

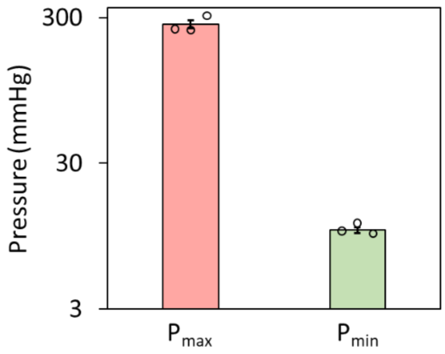

Supplementary Figure 5: Characterization of the maximum tolerable pressure (P_{max}) and minimum required pressure (P_{min}). Error bars, mean ± s.e (n = 3 measurements from different devices).

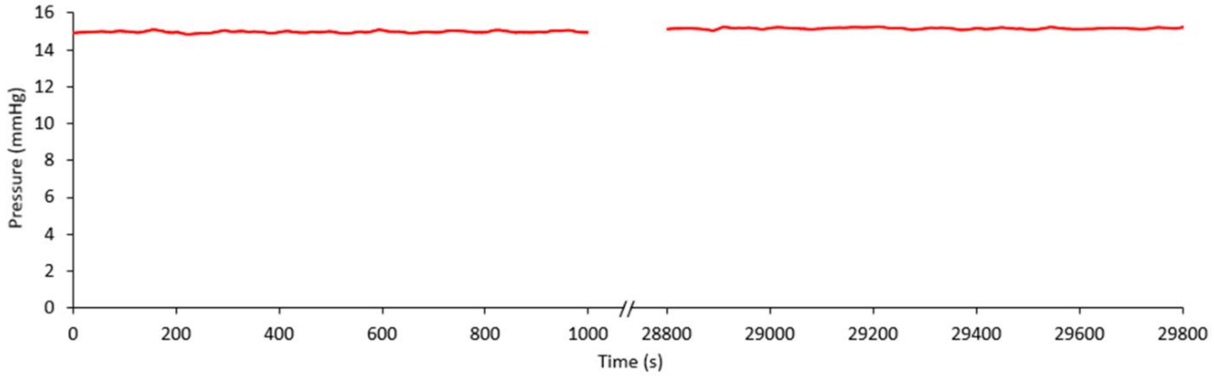

Supplementary Figure 6: Validation of prolonged valve sealing. The maintenance of constant pressure across the valve-gated channel indicates that the channel remained fully sealed by the embedded hydrogel (without suffering from possible dehydration-induced shrinkage effects). Pressure characterization experiment was performed over 8 h, and pressure data was recorded in the first and last 1000 s-period of the experiment to illustrate the unchanged sealing status.

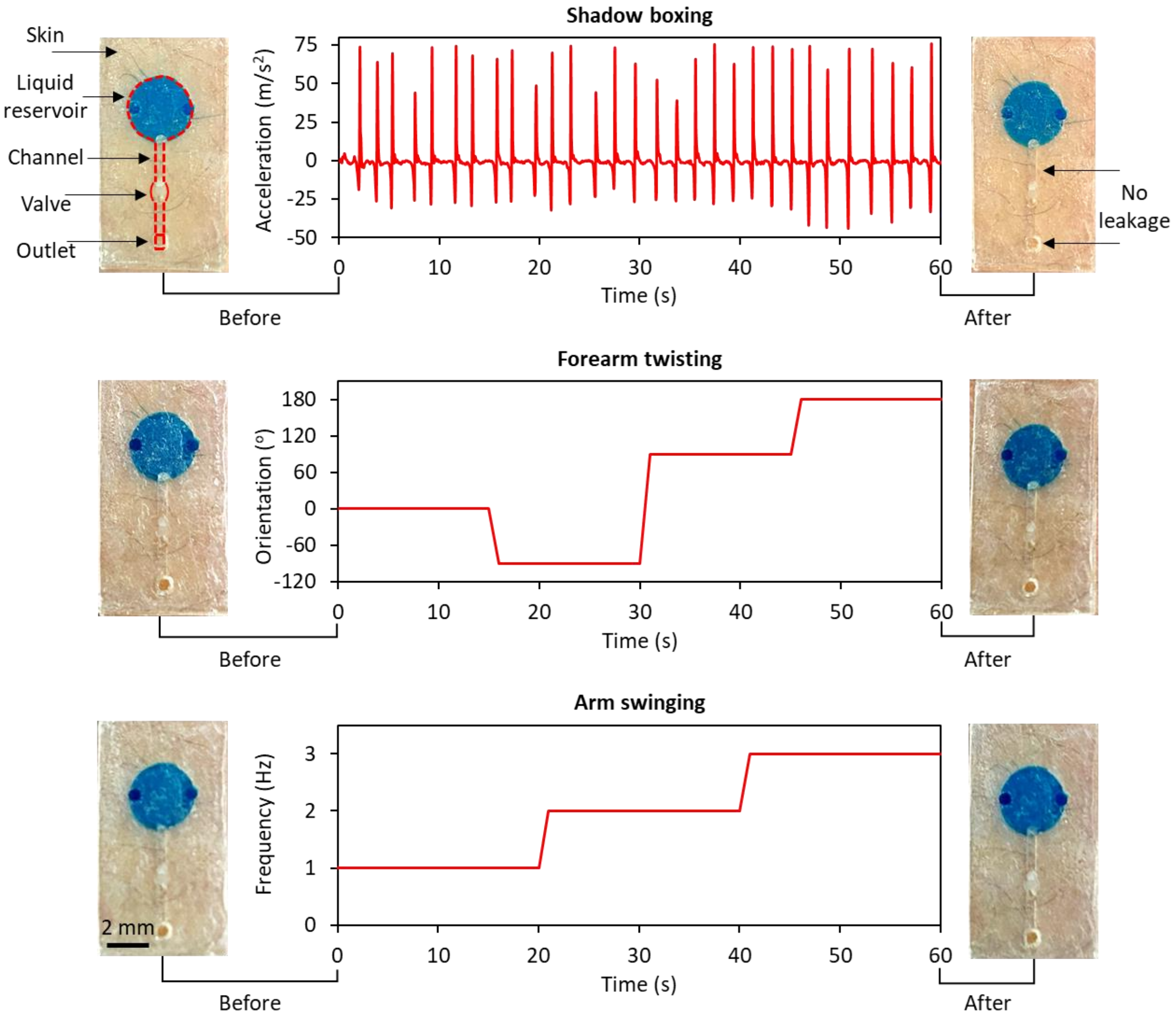

Supplementary Figure 7: On-body validation of valve sealing with a subject—wearing the microfluidic module on the forearm—performing shadow boxing, forearm twisting, and arm swinging at different acceleration levels, orientations, and frequencies, respectively. Optical images of the microfluidic module before/after the activities demonstrate the leakage-free preservation of the compartmentalized blue-dyed sample, illustrating the device robustness under routine user motion.

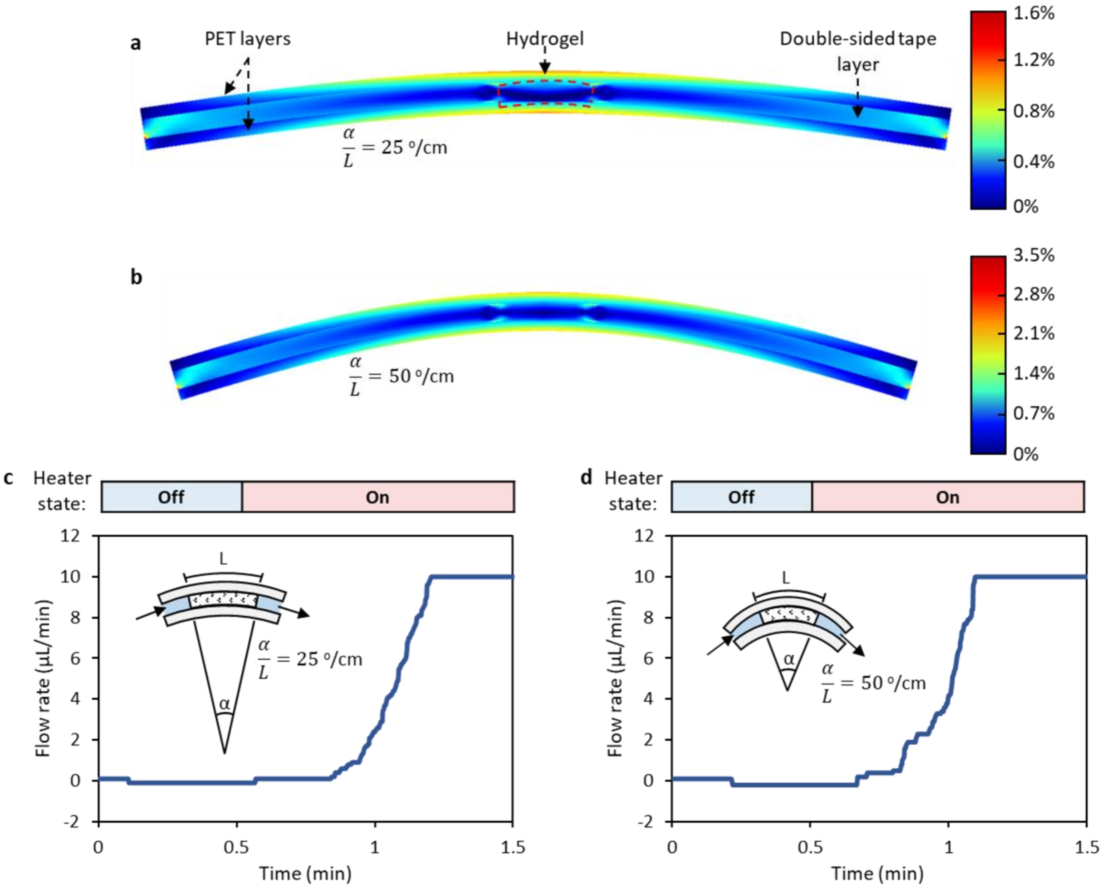

Supplementary Figure 8: a-b COMSOL-simulated strain profile (cross-view) of a flexible microfluidic valve, under two different device bending curvatures: **a** α/L = 25 °/cm and **b** α/L = 50 °/cm. The hydrogel embodiment sustains minimal strain, as it is located at the neutral plane. Hydrogel valve: 1 mm in length, 170 µm in height. **c-d** Experimental validation of fluid valving under two device bending curvatures: **c** α/L = 25 °/cm and **d** α/L = 50 °/cm. The valve is activated after 0.5 min.

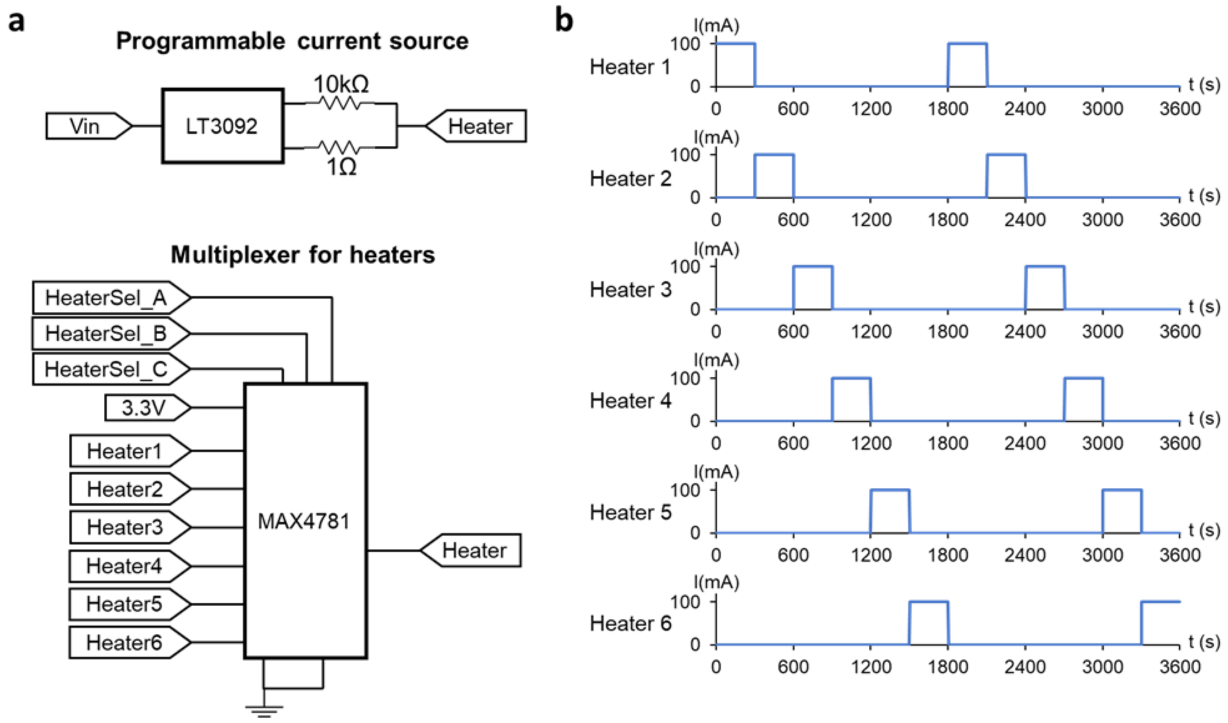

Supplementary Figure 9: a Schematic diagram of the actuation circuit, including the programmable current source and multiplexer (for microheaters) circuitries. **b** The measured current through six electrical resistive microheaters upon the periodic and sequential activation/deactivation of the microheater array (resistive load: 25 Ω, connected at the output of each of the actuation channels).

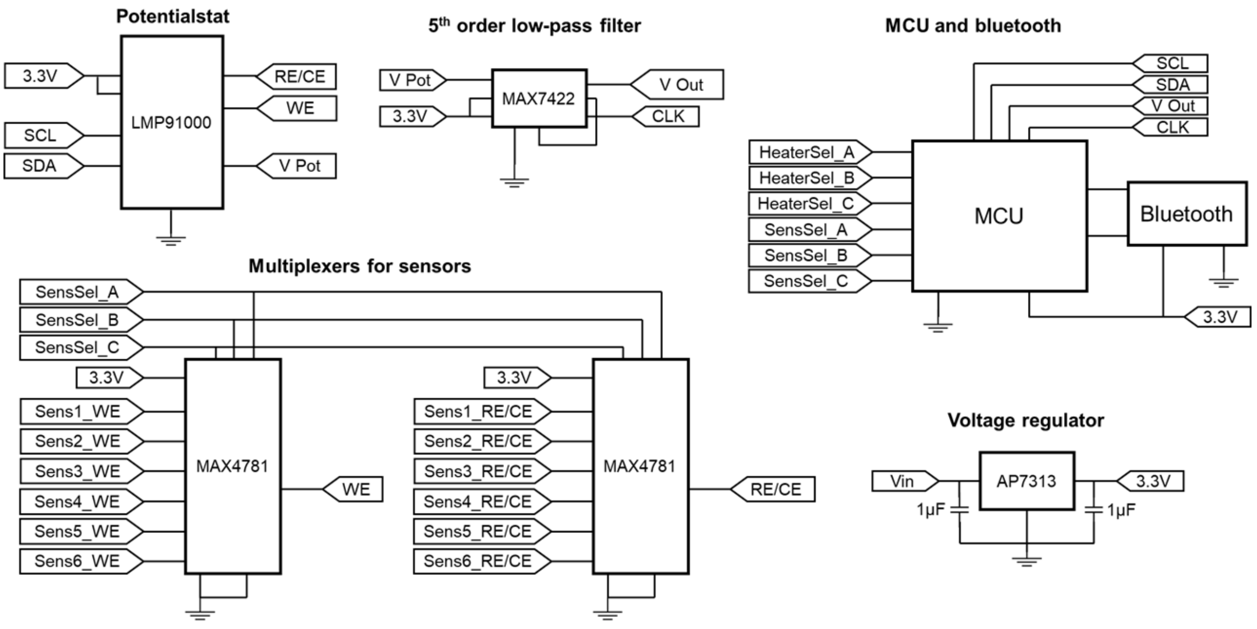

Supplementary Figure 10: Schematic diagrams of the sensing (consisting of potentiostat and LPF), MCU, wireless transmission (Bluetooth), and power regulating circuits.

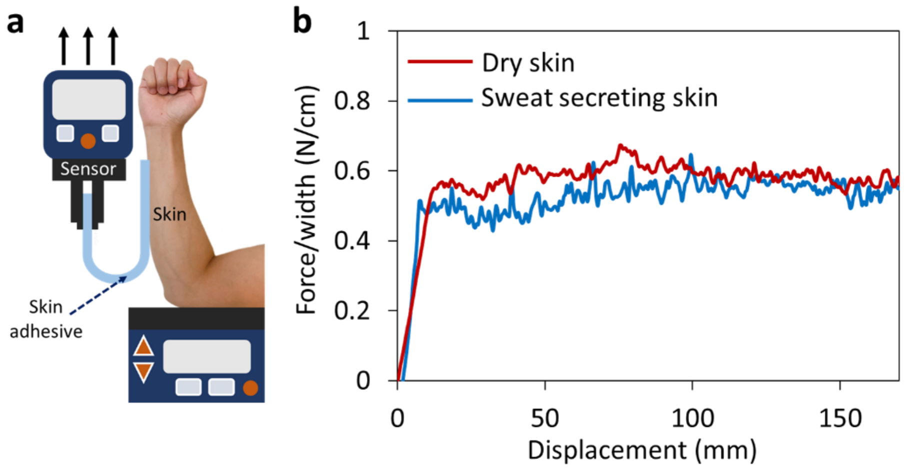

Supplementary Figure 11: a. Setup for the 180° peeling adhesion force characterization. **b**. Characterization of the adhesion force between the skin-adhesive tape (bottom layer of the developed microfluidic device) and skin interface (performed on dry and exercise-induced sweat secreting skin). The results illustrate that the adhesion forces are of similar strengths in both scenarios.

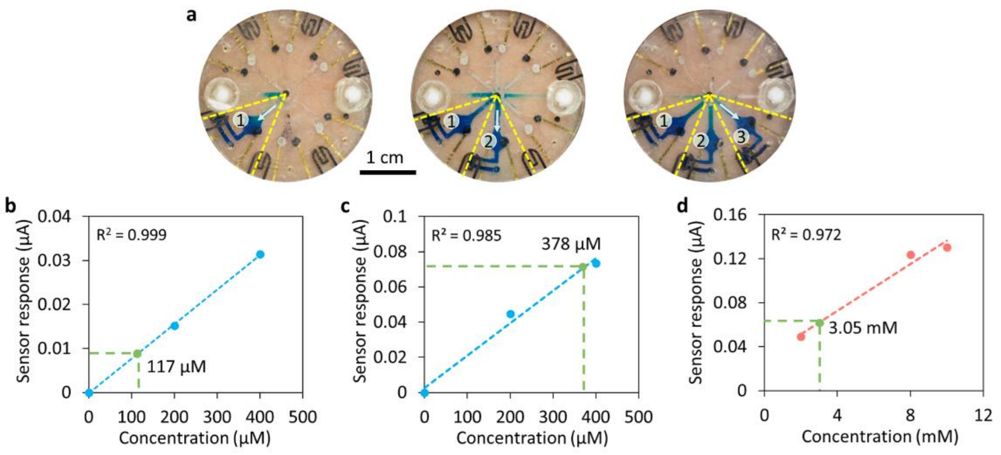

Supplementary Figure 12: a intermittent sample compartmentalization *via* sequential on-body valving (using blue dyes for visualization). **b-d** On-body sweat glucose (**b**, **c**) and lactate (**d**) sensor readouts and the corresponding calibration curves. Sweat glucose readouts in (**b**) and (**c**) were obtained before and after beverage intake, respectively.

a

Electronic components	Operation current	Required capacity
Potentiostat chip	10 μA	30 μA·h
MCU	4 mA	12 mA·h
Bluetooth	5 mA	15 mA·h
Heater	100 mA	20 mA·h

b

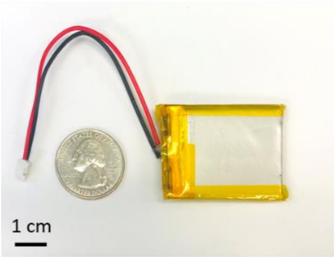

Supplementary Figure 13: a Power requirements for key electronic components. **b** Photograph of a rechargeable lithium-ion polymer battery module used to power the FPCB module (placed next to the Washington quarter for visual comparison).

Supplementary Tables:

Supplementary Table 1: Young’s modulus of the device materials

Material	Young’s Modulus
PET	2.7 GPa
Double-sided tape	450 kPa
Hydrogel	5 kPa
Polyimide (FPCB)	2.5 GPa

Supplementary Table 2: Chemical composition of the enzymatic sensing interfaces

Chemical name	Deposition method	Function/Role
Gold (Au)	E-beam deposition	Electron transfer
Platinum nanoparticle (PtNP)	Electrodeposition	Electrochemical catalyst/electron transfer
Poly(*m*-phenylenediamine)	Electrodeposition	Permselective membrane
Enzymatic layer<br>(Glucose- and lactate-oxidase)	Drop casting	Glucose catalyst<br>(enzyme activity: 100-250 units/mg)<br><br>Lactate catalyst<br>(enzyme activity: ~100 units/mg)
Polyvinyl chloride (PVC)	Drop casting	Diffusion limiting layer<br>(only for the lactate sensor)

References

1. Olsson, B. *et al*. Theory and application of diffusion-limited amperometric enzyme electrode detection in flow injection determination of glucose. *Anal. Chem.* **6**, 1046-1052 (1986).
2. Squires, T. M., Messinger, R. J. & Manalis, S. R. Making it stick: convection, reaction and diffusion in surface-based biosensors. *Nature biotechnology* **26**, 417–426 (2008).
3. Shen, Z. et al. Exploring thermal reversible hydrogels for stem cell expansion in three-dimensions. Soft Matter vol. 8 7250 (2012).
